# Mendelian randomisation reveals Sodium-glucose Cotransporter-1 inhibition's potential in reducing Non-Alcoholic Fatty Liver Disease risk

**DOI:** 10.1093/ejendo/lvad068

**Published:** 2023-06-21

**Authors:** Laurence J Dobbie, Daniel J Cuthbertson, Theresa J Hydes, Uazman Alam, Sizheng Steven Zhao

**Affiliations:** Department of Cardiovascular and Metabolic Medicine, Institute of Life Course and Medical Sciences, University of Liverpool, Liverpool, L7 8TX, United Kingdom; Department of Diabetes & Endocrinology, Guys Hospital, Guy's and St Thomas’ NHS Foundation Trust, London, SE1 9RT, United Kingdom; Department of Cardiovascular and Metabolic Medicine, Institute of Life Course and Medical Sciences, University of Liverpool, Liverpool, L7 8TX, United Kingdom; University Hospital Aintree, Liverpool University Hospitals NHS Foundation Trust, Liverpool, L9 7JR, United Kingdom; Department of Cardiovascular and Metabolic Medicine, Institute of Life Course and Medical Sciences, University of Liverpool, Liverpool, L7 8TX, United Kingdom; University Hospital Aintree, Liverpool University Hospitals NHS Foundation Trust, Liverpool, L9 7JR, United Kingdom; Department of Cardiovascular and Metabolic Medicine, Institute of Life Course and Medical Sciences, University of Liverpool, Liverpool, L7 8TX, United Kingdom; University Hospital Aintree, Liverpool University Hospitals NHS Foundation Trust, Liverpool, L9 7JR, United Kingdom; Centre for Epidemiology Versus Arthritis, Division of Musculoskeletal and Dermatological Science, School of Biological Sciences, Faculty of Biological Medicine and Health, The University of Manchester, Manchester Academic Health Science Centre, Manchester, M13 9PT, United Kingdom

**Keywords:** sodium-glucose cotransporter, NAFLD, liver transaminases, SGLT1, glycated hemoglobin, type 2 diabetes

## Abstract

Non-alcoholic fatty liver disease (NAFLD) has no approved pharmacological treatments. Sodium-glucose cotransporter (SGLT)-1 is a glucose transporter that mediates small intestinal glucose absorption. We evaluated the impact of genetically proxied SGLT-1 inhibition (SGLT-1i) on serum liver transaminases and NAFLD risk. We used a missense variant, rs17683430, in the SLC5A1 gene (encoding SGLT1) associated with HbA1c in a genome-wide association study (*n* = 344 182) to proxy SGLT-1i. Outcome genetic data comprised 1483 NAFLD cases and 17 781 controls. Genetically proxied SGLT-1i was associated with reduced NAFLD risk (OR 0.36; 95%CI 0.15, 0.87; *P* = .023) per 1 mmol/mol HbA1c reduction, and with reductions in liver enzymes (alanine transaminase, aspartate transaminase, gamma-glutamyl transferase). Genetically proxied HbA1c, not specifically via SGLT-1i, was not associated with NAFLD risk. Colocalisation did not demonstrate genetic confounding. Overall, genetically proxied SGLT-1i is associated with improved liver health, this may be underpinned by SGLT-1-specific mechanisms. Clinical trials should evaluate the impact of SGLT-1/2 inhibitors on the prevention and treatment of NAFLD.

**Significance**

There is a current epidemic of NAFLD, driven by obesity and type 2 diabetes (T2D). There are currently no approved pharmacological therapies for NAFLD. Using genome-wide association study data we analyzed how genetically proxied inhibition of SGLT-1 (a small intestinal transporter mediating glucose absorption) impacts liver enzymes and NAFLD risk. Using two-sample Mendelian randomization we demonstrate that genetically proxied SGLT-1i reduces liver enzymes and NAFLD risk via a reduction in HbA1c. The significant effect size on NAFLD risk via HbA1c reduction which is mediated through SGLT-1i, but not overall HbA1c, suggests the importance of SGLT-1i-specific mechanisms. Overall, the clinical efficacy of dual inhibition of SGLT-1/2 in the treatment and prevention of NAFLD warrants evaluation in well-designed clinical trials.

## Introduction

Non-alcoholic fatty liver disease (NAFLD) is a metabolic disease, that commonly co-exists with type 2 diabetes (T2D) and obesity. NAFLD is characterized by hepatic accumulation of triglycerides (steatosis), which is a subset of people, progresses to non-alcoholic steatohepatitis (NASH) and eventually liver fibrosis.^1,2^ T2D is a key factor in NAFLD pathophysiology; insulin resistance contributes toward elevated serum-free fatty acid concentrations which can be deposited within the liver. NAFLD is highly prevalent, with up to 1 in 3 individuals living with the condition.^3^ This is concerning, given that NAFLD-associated liver fibrosis occurs in up to 40% of affected people, with NAFLD projected to become the most frequent indication for liver transplantation.^2^ Current therapeutic strategies are sub-optimal, focusing on lifestyle intervention including weight loss.^1^ A novel NAFLD therapeutic paradigm is urgently required.

NAFLD development is partly driven by glucotoxicity.^4-6^ Clinical trials of sodium-glucose cotransporter-2 inhibitors (SGLT-2i), which inhibit renal tubular glucose absorption, demonstrate that in patients with T2D and NAFLD, SGLT-2i improves liver enzymes and reduces liver fat.^7-9^ SGLT-1 is a potent mediator of intestinal glucose absorption that contributes to NAFLD through increased glucose flux to the liver. Thus, SGLT-1 may reduce NAFLD risk via improved glycaemic control, increased residual gastrointestinal (GI) tract glucose contributing towards favorable neuroendocrine hormone levels, or reduced post-prandial glucose load.^10-12^ Clinically, the use of the dual SGLT-1/2 inhibitor licogliflozin for 12 weeks reduced serum alanine transaminase (ALT) concentration in participants with NASH, but this randomized trial did not include participants across the NAFLD-disease spectrum.^13^

Further evaluation of the clinical effects of SGLT-1i in NAFLD is justified. Natural variation in the genes that encode protein drug targets can offer insight into mechanism-based efficacy and safety.^14^ Such genetic instrumental variable analysis, or Mendelian randomization (MR), is more robust against confounding than traditional epidemiologic designs.^15^ Since genetic variants are randomly allocated at conception, MR can be conceptualized as a quasi-randomized natural experiment comparing NAFLD risk according to levels of genetically proxied SGLT-1 activity. Our aim was to use MR to investigate the effect of genetically proxied SGLT-1i on NAFLD risk and liver enzyme levels.

## Methods

All summary statistics from prior genome-wide association studies (GWAS) are publicly available and had previously received appropriate patient consent and ethical approval. Full details are available in the original publications.^16,17^ The research complied with the declaration of Helsinki. Methods S1 provides details on genetic proxies for SGLT1i and HbA1c as well as a genetic association for outcome measures.

### Statistical analysis and MR assumptions

We used the Wald ratio method to estimate the association of genetically proxied SGLT1i with each outcome, whereby the exposure-outcome estimate is derived from the variant-outcome association divided by the variant-exposure association.

Analysis using multiple instruments for genetically predicted HbA1c was performed using the fixed-effect inverse-variance weighted (IVW) method.^18^

Valid instrumental variables are defined by three assumptions,^19^ which we interrogated as follows. First, variants must be associated with the exposure of interest. F statistic was derived using the chi-square approximation.^20^ F statistic >10 is suggestive of adequate instrument strength.^21^ Second, the variants should share no common cause with the outcome (ie, no unmeasured confounders). This assumption is not empirically verifiable, although before a study of *SLC5A1* missense variants showed no association with smoking, alcohol, or total energy intake.^22^ We also tried to minimize bias arising from underlying population structure through the use of European ancestry populations. We also performed colocalization analysis to examine possible genetic confounding through linkage disequilibrium (LD) using default prior probabilities (ie, 10^−4^, 10^−4^, and 10^−5^ for a variant within the *SLC5A1* genomic locus being associated with the exposure trait, outcome trait, or both traits, respectively). Third, variants should not affect the outcome except through the risk factor. The use of a missense variant with plausible biology reduces the risk of this bias. Analyses were performed in R using the *TwoSampleMR* and *coloc* packages.^23,24^

## Results

Three protein-coding variants in high LD (*r*^2^ = 1) were identified in the *SLC5A1* gene. The lead variant, rs17683430 (F statistic 59), was used to instrument SGLT1i. Table 1 details the GWAS data included for analysis.

**Table 1.** Summary of genome-wide association studies for analyses.

Study	*N* (case/controls)	Phenotype (definition/unit)	Ancestry
Glycated hemoglobin (HbA1c) (UKBB)^16^	344 182	1 mmol/mol	EUR
NAFLD (Anstee et al)^17^	1483 NAFLD/17781 Control	Liver biopsy proven NAFLD	EUR
ALT (UKBB)^16^	388 865	U/L	EUR
AST (UKBB)^16^	388 865	U/L	EUR
GGT (UKBB)^16^	388 865	U/L	EUR

Abbreviations: ALT, alanine aminotransferase; AST, aspartate aminotransferase; EUR, European; GGT, gamma-glutamyl transferase; NAFLD, non-alcoholic fatty liver disease; U/L, units/litre; UKBB, UK Biobank; EUR, European.

In the primary analysis, genetically proxied SGLT1i was associated with a 64% reduction in risk of NAFLD (odds ratio [OR]: 0.36; 95%CI 0.15, 0.87; *P* = .023) per 1 mmol/mol HbA1c reduction. Genetically proxied SGLT1i was associated with reductions in ALT (−0.98 U/L, 95%CI −1.59, −0.37, *P* < .01), AST (−0.58 U/L, 95%CI −1.05, −0.10, *P* = .02), and gamma-glutamyl transferase (GGT) (−3.73 U/L, 95%CI −5.61, −1.85, *P* < .01).

**Figure 1.**
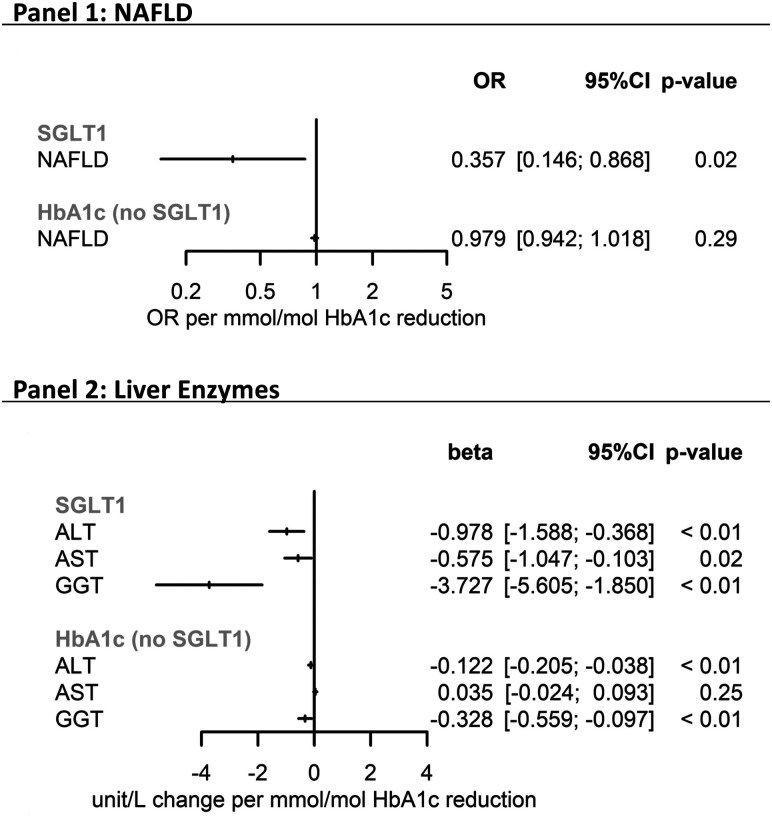
Forest plots of Mendelian randomisation results. Panel 1: NAFLD; forest plot depicting NAFLD risk on a linear scale. Panel 2: liver enzymes; forest plot depicting liver enzymes on a logarithmic scale. NAFLD, non-alcoholic fatty liver disease; U/L, units/litre; ALT, alanine aminotransferase; AST, aspartate aminotransferase; GGT, gamma-glutamyl transferase; OR, odds ratio.

In comparison, genetically proxied HbA1c (instrumented using 186 single nucleotide polymorphisms (SNPs) for Liver Enzymes and 155 SNPs for NAFLD), not specifically via SGLT-1i, was associated with reduced ALT and GGT, but not with AST or NAFLD risk (Figure 1). Associations with ALT and GGT were directionally discordant in pleiotropy robust sensitivity analyses, suggesting the presence of bias. (Table S1).

### Colocalization

For NAFLD, the probability of colocalization conditional on the presence of a causal variant for the outcome was 89%. For ALT, aspartate transaminase (AST) and gamma-glutamyl transferase (GGT) equivalent probabilities were 87%–89% (Table S2). These results suggest that the MR estimates for the effect of SGLT1i on NAFLD, ALT, AST, and GGT are unlikely to be confounded by a variant in LD. Locus plots are displayed in Figures S1–S4.

## Discussion

We conducted a two-sample MR study to investigate whether SGLT-1i may be a potential pharmacological therapy for patients with NAFLD. Genetically proxied SGLT-1i significantly reduces NAFLD risk and liver enzymes. The estimates for HbA1c reduction via SGLT-1i were greater than for overall HbA1c (not via SGLT-1), indicating additional mechanisms specific to SGLT-1 may be important. We also show a more significant association of SGLT-1i with a reduction in serum ALT rather than AST concentration, a pattern consistent with liver fat reduction.^25^ Given the emerging evidence of SGLT-2i in the treatment of NAFLD,^7,8,25^ clinical trials evaluating dual SGLT1/2 inhibitors are needed to investigate its therapeutic potential and preventative effects for NAFLD.

Novel pharmacotherapies for NAFLD are urgently needed.^26^ SGLT-2i is a potential option: in the E-LIFT (effects of empagliflozin on liver fat content in patients with type 2 diabetes) trial empagliflozin significantly reduced liver fat and improved liver enzymes.^7^ Pooled data in the EMPA-REG outcome study (empagliflozin cardiovascular outcome event trial in type 2 diabetes mellitus patients) demonstrated that empagliflozin reduced ALT independent of body weight.^25^ However, efficacy and safety may be limited in those with renal impairment. Our data highlight that SGLT-1i reduces NAFLD risk and improves liver enzymes. These results are in keeping with phase 2 data in patients with NASH, where licogliflozin, a dual SGLT1/2 inhibitor, potently reduced liver enzymes.^13^ The highest dose, 150 mg, also reduced liver fat. Further data are needed to evaluate Licogliflozin in patients with NAFLD fibrosis. Overall, the addition of SGLT-1i in combination with SGLT-2i may have a synergistic effect in improving liver health. Dual SGLT-1/2 inhibitors warrant an evaluation in clinical trials of participants with NAFLD.

### Mechanistic underpinning

**Figure 2.**
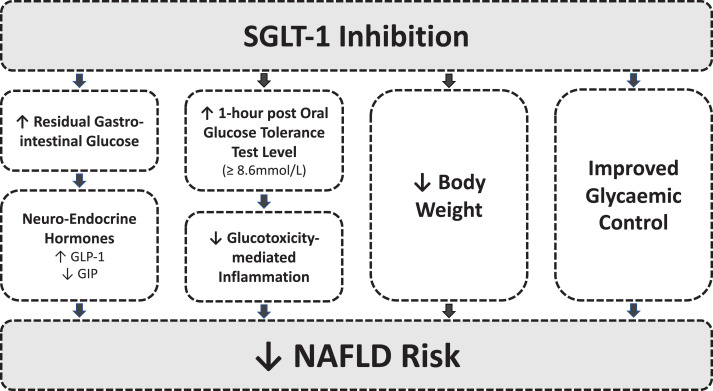
Graphical summary delineating how SGLT-1 inhibition may impact NAFLD development. GI, gastrointestinal; GLP-1, glucagon-like-peptide 1; GIP, gastrointestinal polypeptide; NAFLD, non-alcoholic fatty liver disease; OGTT, oral-glucose tolerance test; SGLT-1, sodium-glucose cotransporter 1.

Our data shows that HbA1c reduction via genetically proxied SLGT-1i reduces NAFLD risk and improves liver enzymes. This pattern was not reflected in overall HbA1c reduction meaning SGLT-1i may act through HbA1c-independent mechanisms. SGLT-1 modulates entero-endocrine hormone regulation, for instance, reduced SGLT-1-mediated glucose absorption leads to residual GI glucose which stimulates Glucagon-like Peptide-1 (GLP-1) and inhibits gastrointestinal polypeptide (GIP).^12,27^ Sotagliflozin, a dual SGLT1/2i, increases GLP-1 and reduces GIP in a pattern consistent with reduced liver adiposity.^10,28-30^ Chronic hyperglycemia also contributes to hepatic fat accumulation. A raised 1-hour post-oral glucose tolerance test (OGTT) level (≥8.6 mmol/L) is implicated in NAFLD risk via glucotoxicity-mediated inflammation. Small intestinal SGLT-1 abundance correlates with raised 1-hour glucose level post-OGTT, but not with fasting glucose or 2-hour OGTT level.^5,6,11,31^ Increased 1-hour post-OGTT glucose level enhances the risk of NAFLD and liver enzyme derangement, therefore SGLT-1i may reduce 1-hour post-OGTT glucose level and potentially NAFLD risk.^32-34^ SGLT-1 expression is higher in those with obesity, with obesity being a risk factor for NAFLD.^35-37^ SGLT-1i may be useful in the phenotype of patients with NAFLD, obesity, and T2DM.^35^ Overall, SGLT-1i inhibition may reduce NAFLD risk by pleiotropic effects including: (1) reduction in HbA1c, (2) modulation of neuroendocrine signaling, (3) body weight reduction, and (4) reduced 1-hour OGTT glucose level (Figure 2).^10-12^ Further evaluation is required.

### Limitations

A key limitation is that MR provides an association of genetically predicted SGLT-1i over a lifetime, meaning effect estimates may be larger than quantified in adult life studies.^14^ Second, the risk factors for disease onset may not be equivalent to those for disease severity or prognosis. Therefore, our results are more applicable to NAFLD prevention. Third, it is important to note that the effects of genetic variation on SGLT1 levels cannot be directly compared to the effects of pharmacological inhibition. Differences in exposure duration and tissue specificity may also play a role. Fourth, as with all MR studies, the assumptions made in instrumental variable analysis cannot be empirically verified. Although we conducted sensitivity analyses to address potential sources of bias, it is still possible that pleiotropy or confounding may affect our estimates. rs17683430 is associated with the expression of several genes; however, the most strongly associated expression quantitative loci relates to SLC5A1, suggesting that any pleiotropic effects may be modest. Finally, our study population consisted only of participants of European ancestry.

## Conclusions

We report that genetically proxied SGLT-1i reduces NAFLD risk and improves liver enzymes in a population with a low prevalence of diabetes. Our data also point toward this risk reduction being partially mediated via SGLT-1-specific mechanisms. SLGT-1i is also associated with ALT to a greater extent than AST, a transaminase pattern consistent with liver fat reduction. Overall, clinical trials should investigate SGLT1/2 inhibitors in the NAFLD-disease spectrum.

## Supplementary Material

lvad068_Supplementary_Data

## Data Availability

All summary statistics used in this study are publicly available, with relevant citations detailed.
