# Supplementary material for: Mendelian randomisation reveals Sodium-glucose Cotransporter-1 inhibition's potential in reducing Non-Alcoholic Fatty Liver Disease risk
Source: Eur J Endocrinol. 2023 Jun 21;188(6):K33–7. doi: 10.1093/ejendo/lvad068 (PMC11188739; doi:10.1093/ejendo/lvad068)

**Supplementary Materials**

**Mendelian Randomisation reveals SGLT-1 inhibition’s potential in Reducing NAFLD Risk**

**Laurence J Dobbie^1,2^, Daniel J Cuthbertson^1,3,*^, Theresa J Hydes^1,3^, Uazman Alam^1,3^, Sizheng Steven Zhao^4^**

^1^Department of Cardiovascular and Metabolic Medicine, Institute of Life Course and Medical Sciences, University of Liverpool, Liverpool, UK.

^2^Department of Diabetes & Endocrinology, Guys Hospital, Guy's and St Thomas' NHS Foundation Trust, London, United Kingdom

^3^ University Hospital Aintree, Liverpool University Hospitals NHS Foundation Trust, Liverpool, UK

^4^Centre for Epidemiology Versus Arthritis, Division of Musculoskeletal and Dermatological Science, School of Biological Sciences, Faculty of Biological Medicine and Health, The University of Manchester, Manchester Academic Health Science Centre, Manchester, UK.

*: Corresponding Author

Prof Daniel J Cuthbertson

Department of Cardiovascular and Metabolic Medicine, Institute of Life Course and Medical Sciences, University of Liverpool, Liverpool, UK.

**Supplementary Methods 1**

Genetic proxies for SGLT1i

We chose HbA1c as the biomarker of SGLT1i action because of the established effect of SGLT inhibition on glycaemic control in randomised controlled trials (RCTs) of T2DM.[1–4] We described instrument selection for SGLT1i previously [5]; we selected protein-coding variants within the *SLC5A1* gene (build 37; chromosome 22: 32439019-32509016) that were associated with HbA1c (p<5x10^-8^) and uncorrelated (linkage disequilibrium threshold of r^2^<0.1). Genetic associations were derived from 344,182 participants of European ancestry in the UK Biobank study.[6] Approximately 5% of this population had T2DM at enrolment.

Genetic proxies for HbA1c

We investigated whether genetically predicted HbA1c overall (rather than specifically through SGLT1i) is associated with NAFLD. We instrumented HbA1c modification using variants throughout the genome, except variants from the *SLC5A1* gene ± 300 kilobases that had associations with HbA1c at p<5x10^-8^ and r^2^<0.001. This stricter correlation was used because variants were selected from throughout the genome, rather than a single gene locus.

Genetic association for outcomes

Genetic association data for NAFLD was obtained from the GWAS by Anstee et al. NAFLD cases underwent liver biopsy for diagnosis.[7] Data for Alanine aminotransferase (ALT), Aspartate aminotransferase (AST) and Gamma glutamyl transferase (GGT) were taken from GWAS of the UK Biobank study (field 30620, 30650, 30730, respectively, each in units/L).[6]

**References**

[1] Bhatt DL, Szarek M, Pitt B, Cannon CP, Leiter LA, McGuire DK, et al. Sotagliflozin in Patients with Diabetes and Chronic Kidney Disease. N Engl J Med 2021;384:129–39. https://doi.org/10.1056/NEJMOA2030186.

[2] Bhatt DL, Szarek M, Steg PG, Cannon CP, Leiter LA, McGuire DK, et al. Sotagliflozin in Patients with Diabetes and Recent Worsening Heart Failure. N Engl J Med 2021;384:117–28. https://doi.org/10.1056/NEJMOA2030183.

[3] Zaccardi F, Webb DR, Htike ZZ, Youssef D, Khunti K, Davies MJ. Efficacy and safety of sodium-glucose co-transporter-2 inhibitors in type 2 diabetes mellitus: systematic review and network meta-analysis. Diabetes Obes Metab 2016;18:783–94. https://doi.org/10.1111/DOM.12670.

[4] Brown E, Heerspink HJL, Cuthbertson DJ, Wilding JPH. SGLT2 inhibitors and GLP-1 receptor agonists: established and emerging indications. Lancet (London, England) 2021;398:262–76. https://doi.org/10.1016/S0140-6736(21)00536-5.

[5] Zhao SS, Rajasundaram S, Karhunen V, Alam U, Gill D. Sodium-glucose cotransporter 1 inhibition and gout: Mendelian randomisation study. Semin Arthritis Rheum 2022;56:152058. https://doi.org/10.1016/j.semarthrit.2022.152058.

[6] Rapid GWAS of thousands of phenotypes for 337,000 samples in the UK Biobank — Neale lab n.d. http://www.nealelab.is/blog/2017/7/19/rapid-gwas-of-thousands-of-phenotypes-for-337000-samples-in-the-uk-biobank (accessed November 12, 2022).

[7] Anstee QM, Darlay R, Cockell S, Meroni M, Govaere O, Tiniakos D, et al. Genome-wide association study of non-alcoholic fatty liver and steatohepatitis in a histologically characterised cohort☆. J Hepatol 2020;73:505–15. https://doi.org/10.1016/j.jhep.2020.04.003.

**Supplementary Table 1: Pleiotropy robust sensitivity analysis for associations between HbA1c and liver outcomes.**

**S1.1: Liver Enzymes**

| **Exposure** | **Outcome** | **Method** | **N SNP** | **Beta** | **95% CI** | | **p** |
| --- | --- | --- | --- | --- | --- | --- | --- |
|  |  |  |  |  | **Lower** | **Upper** |  |
| HbA1c (no SGLT1) | ALT | Inverse variance weighted | 186 | 0.119 | 0.036 | 0.203 | **0.005** |
|  |  | MR Egger | 186 | -0.064 | -0.228 | 0.100 | 0.446 |
|  |  | Weighted median | 186 | 0.006 | -0.053 | 0.065 | 0.835 |
|  |  | Weighted mode | 186 | -0.005 | -0.063 | 0.053 | 0.866 |
|  | AST | Inverse variance weighted | 186 | -0.036 | -0.095 | 0.022 | 0.226 |
|  |  | MR Egger | 186 | -0.148 | -0.264 | -0.033 | **0.013** |
|  |  | Weighted median | 186 | -0.031 | -0.079 | 0.017 | 0.207 |
|  |  | Weighted mode | 186 | -0.042 | -0.113 | 0.030 | 0.256 |
|  | GGT | Inverse variance weighted | 186 | 0.319 | 0.089 | 0.550 | **0.007** |
|  |  | MR Egger | 186 | -0.209 | -0.660 | 0.242 | 0.365 |
|  |  | Weighted median | 186 | 0.266 | 0.086 | 0.446 | **0.004** |
|  |  | Weighted mode | 186 | 0.149 | -0.057 | 0.356 | 0.158 |

**S1.2: NAFLD Risk**

| **Exposure** | **Outcome** | **Method** | **N SNP** | **OR** | **95% CI** | | **p** |
| --- | --- | --- | --- | --- | --- | --- | --- |
|  |  |  |  |  | **Lower** | **Upper** |  |
| HbA1c (no SGLT1) | NAFLD | Inverse variance weighted | 155 | 1.021 | 0.982 | 1.062 | 0.289 |
|  |  | MR Egger | 155 | 1.020 | 0.929 | 1.119 | 0.681 |
|  |  | Weighted median | 155 | 0.991 | 0.935 | 1.050 | 0.758 |
|  |  | Weighted mode | 155 | 0.999 | 0.899 | 1.109 | 0.979 |

NAFLD, Non-Alcoholic Fatty Liver Disease; ALT, Alanine Aminotransferase; AST, Aspartate Aminotransferase; GGT, Gamma-Glutamyl Transferase; 95% CI, 95% Confidence Interval; p, p-value; N SNP, Number Single Nucleotide Polymorphisms

**Supplementary Table 2: Results from Colocalisation analysis**

|  | SNPs | H0 | H1 | H2 | H3 | H4 | H4/(H3+H4) |
| --- | --- | --- | --- | --- | --- | --- | --- |
| NAFLD | 184 | 1.12E-07 | 7.21E-01 | 4.62E-09 | 2.95E-02 | 2.49E-01 | 89% |
| ALT | 314 | 3.28E-08 | 2.52E-01 | 1.05E-08 | 8.02E-02 | 6.68E-01 | 89% |
| AST | 314 | 9.06E-08 | 6.97E-01 | 5.26E-09 | 4.02E-02 | 2.63E-01 | 87% |
| GGT | 314 | 4.53E-08 | 3.48E-01 | 9.79E-09 | 7.47E-02 | 5.77E-01 | 89% |

**
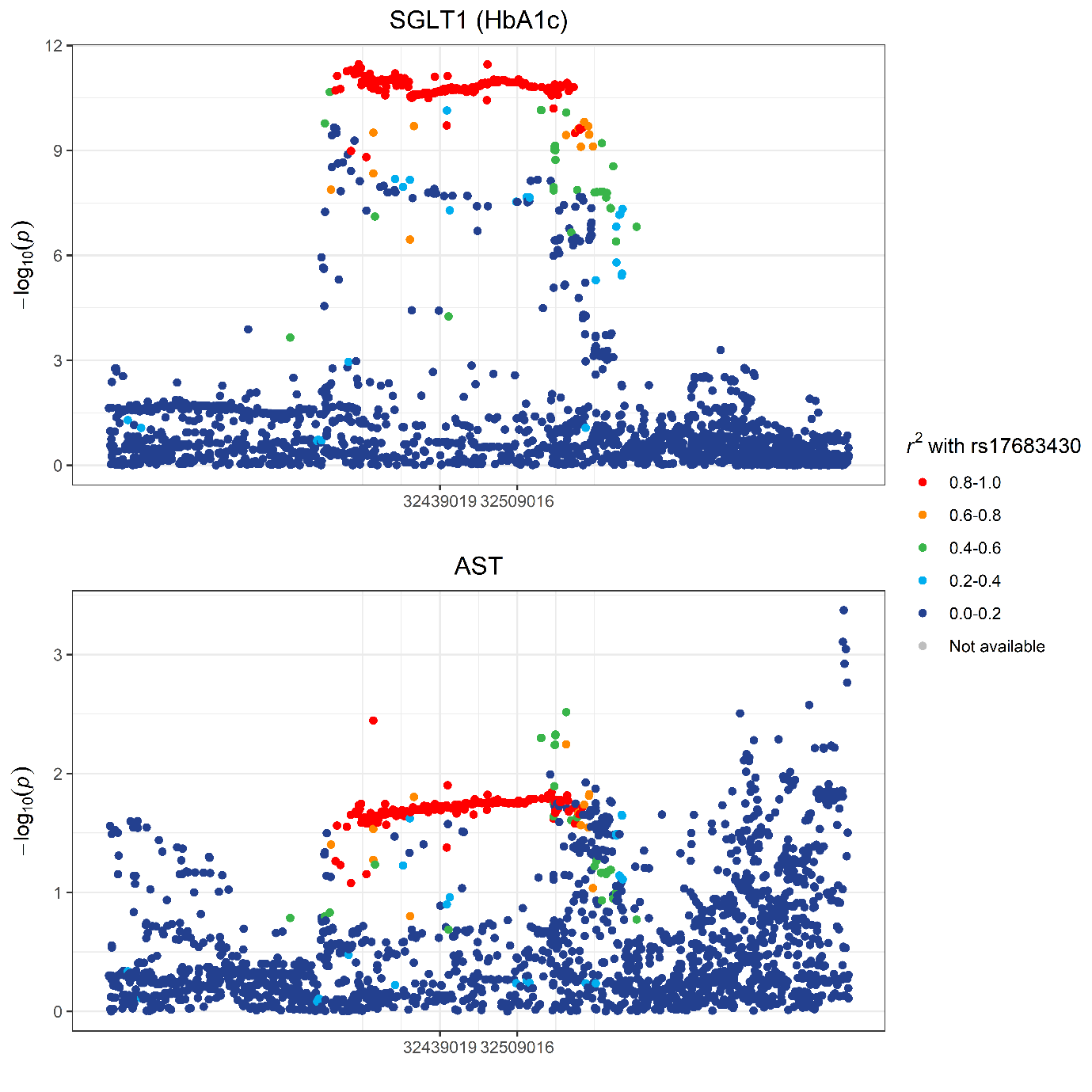
Supplementary Figure 1: Locus Plots of genetic variants associated with AST at the SGLT-1 gene locus**


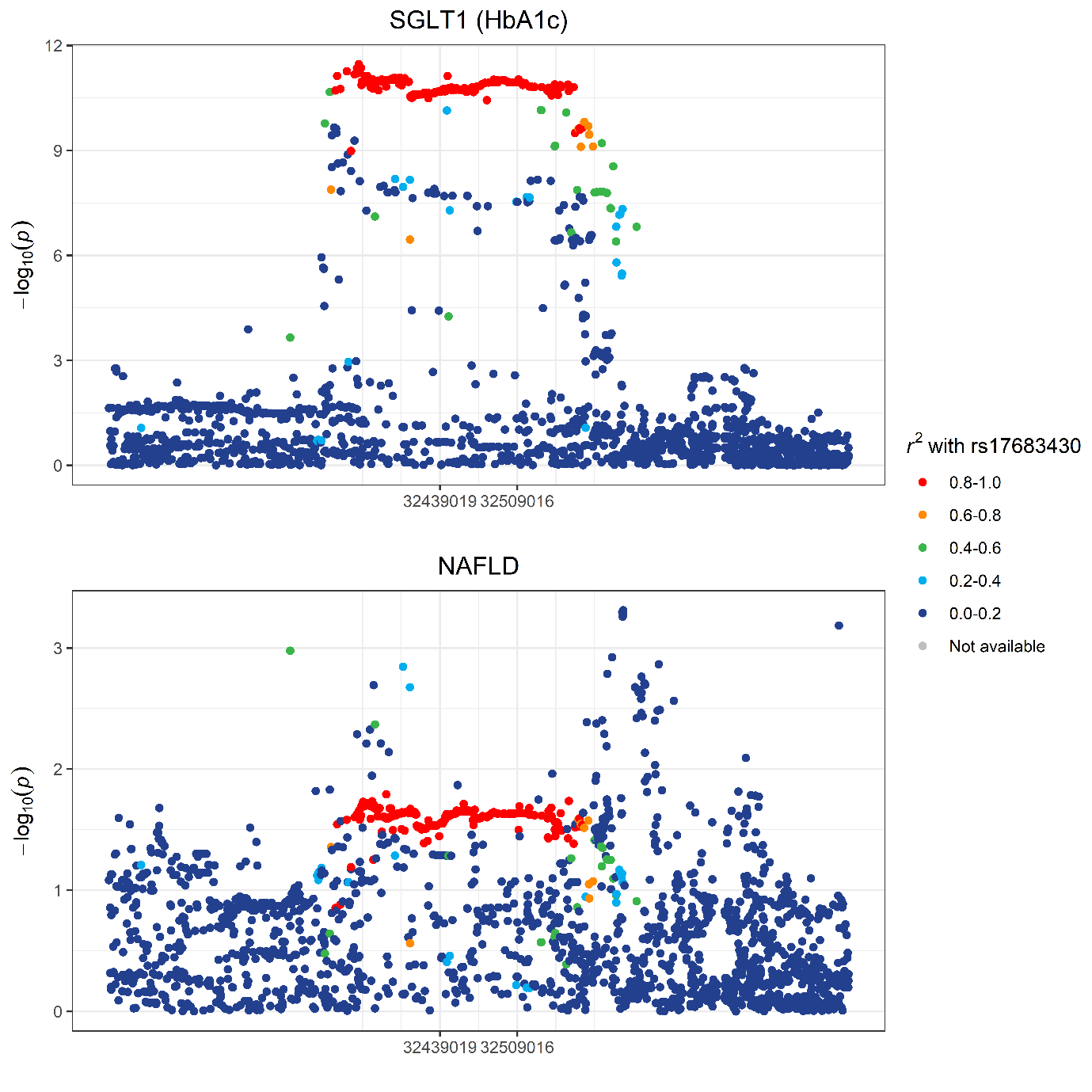
**Supplementary Figure 2:: Locus Plots of genetic variants associated with NAFLD at the SGLT-1 gene locus**


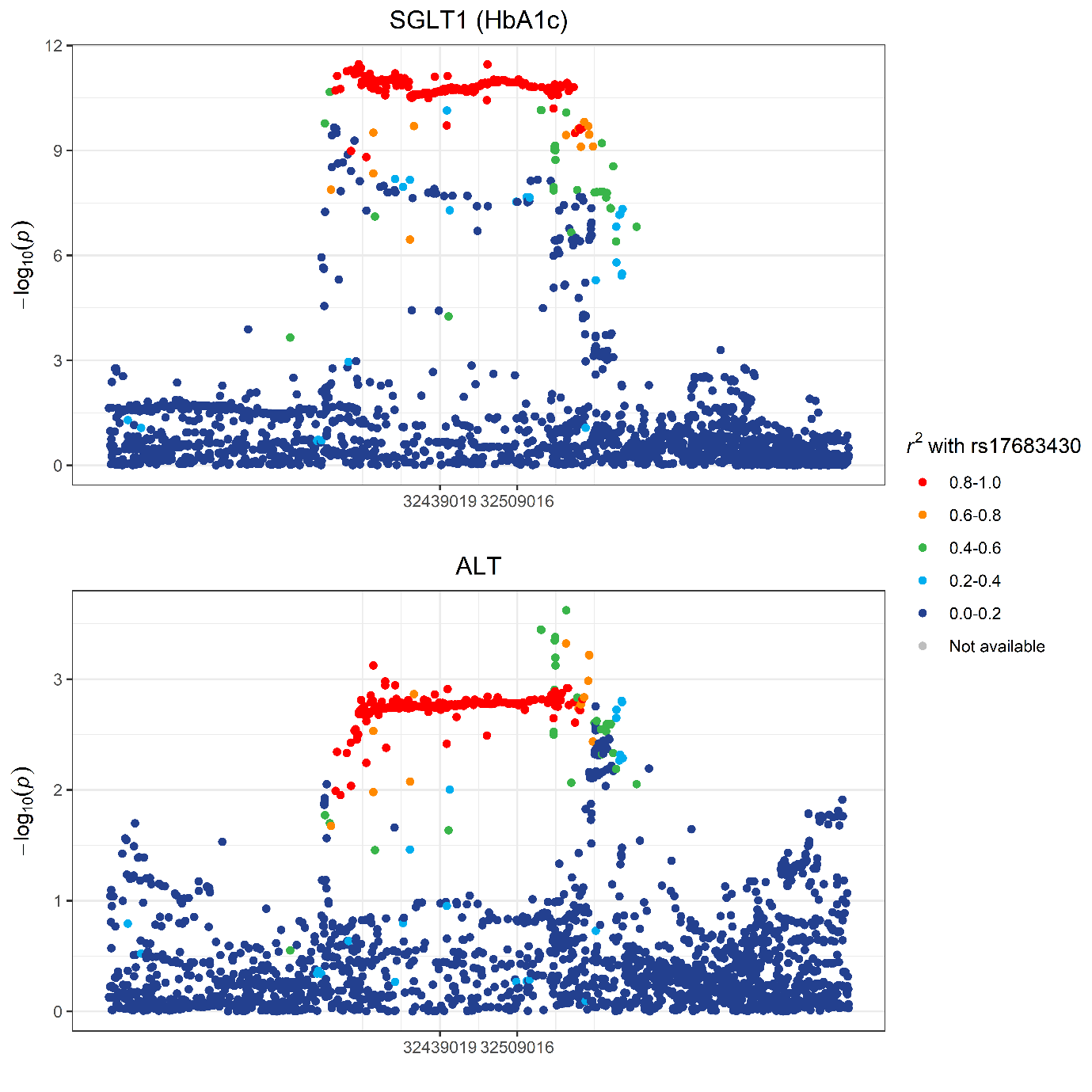
**Supplementary Figure 3: Locus Plots of genetic variants associated with ALT at the SGLT-1 gene locus**

**Supplementary Figure 4: Locus Plots of genetic variants associated with GGT at the SGLT-1 gene locus**


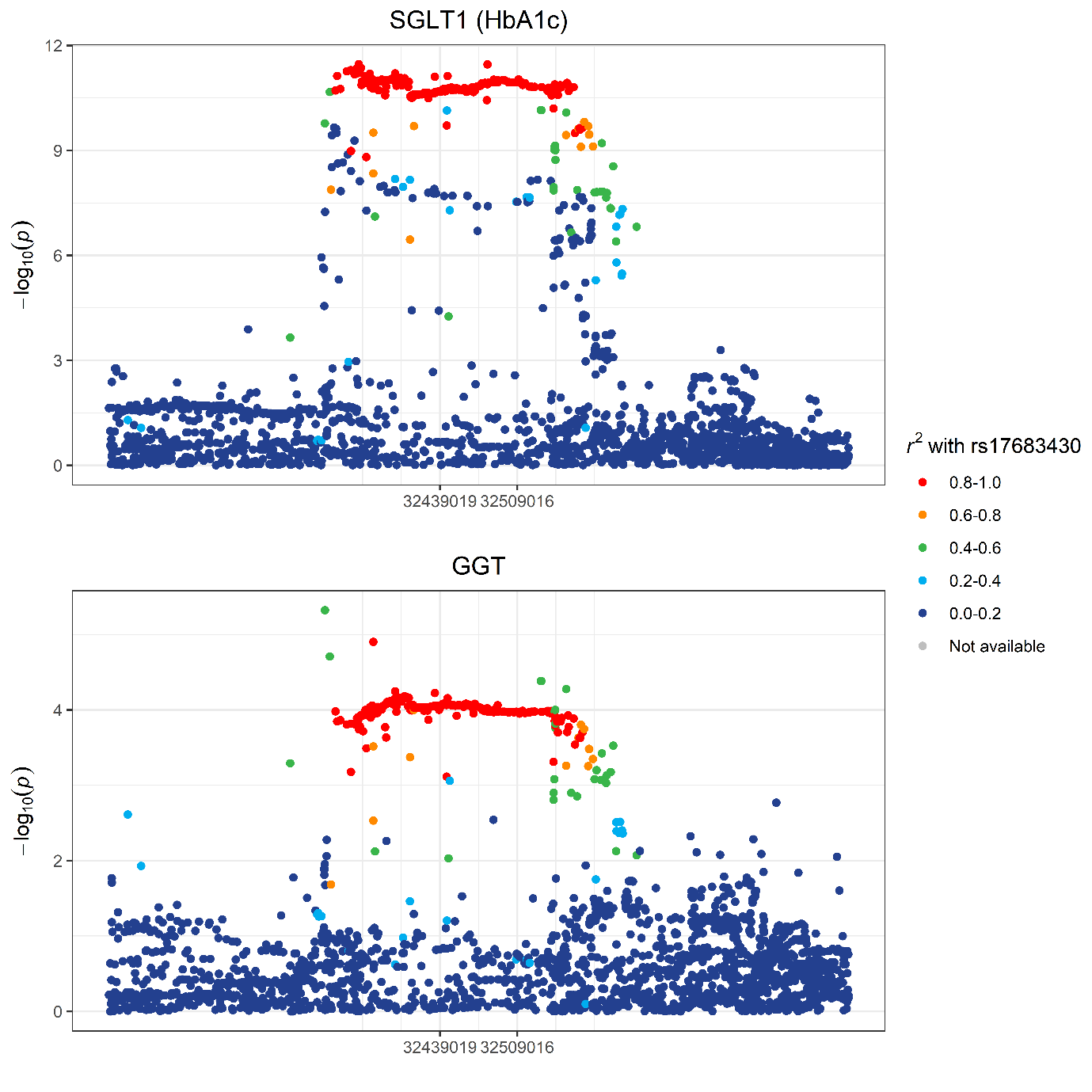

Supplement: lvad068_Supplementary_Data [file lvad068_Supplementary_Data.docx]
